# Seroprevalence of *Toxocara* Infection in Association with Different Risk Factors among Children of 4–12 Years Old Referred to Some Medical Centers in Aras Free Zone, Northwest Iran

**DOI:** 10.18502/ijph.v49i7.3584

**Published:** 2020-07

**Authors:** Elham YOUSEFI, Mohammad Bagher ROKNI, Khosrow HAZRATI TAPPEH, Mehdi MOHEBALI, Shahram KHADEMVATAN, Farzaneh ZAHABIUN, Eshrat Beigom KIA

**Affiliations:** 1. Department of Medical Parasitology and Mycology, School of Public Health, Tehran University of Medical Sciences, Tehran, Iran; 2. Department of Medical Parasitology and Mycology, School of Medicine, Urmia University of Medical Sciences, Urmia, Iran; 3. Cellular and Molecular Research Center, Department of Medical Parasitology and Mycology, Urmia University of Medical Sciences, Urmia, Iran

**Keywords:** Seroprevalence, Toxocariosis, Risk-factors, Children, Iran

## Abstract

**Background:**

Toxocariosis is a parasitic disease caused by the larval stage of *Toxocara* species from dog and cat. It has a worldwide distribution with higher prevalence in children. This study aimed to determine seroprevalence of *Toxocara* infection and its association with some risk factors among children of Aras Free Zone (Jolfa City) in Northwest of Iran.

**Methods:**

Sera were collected from 514 children aged 4–12 yr old attending to some medical centers in the study area from May 2018 to Feb 2019. Anti-*Toxocara* IgG antibodies assay was performed using commercial ELISA kit (Nova Tec, Germany). The seropositivity rate was determined and its association with different demographic criteria and risk factors were statistically analyzed.

**Results:**

The overall seroprevalence was 2.3% (12/514). Risk factors of children’s age group and contact with either pet animals (dog and cat) and/or soil were significantly associated with seropositivity. However, there was not any relationship between *Toxocara* infection and gender of children, place of residency (urban or rural) and their mothers’ education level.

**Conclusion:**

Both girls and boys are at risk of *Toxocara* infection in the study area. Younger age of childhood and contact with sources of infection were important associated factors. More probably, additional criteria are involved in the initiation of infection.

## Introduction

Toxocariosis is one of the most common zoonotic infections with worldwide distribution, especially in countries with low sanitation and hygiene levels (1). The genus *Toxocara* including *Toxocara canis* and less frequently *Toxocara cati* are recognized as causative agents of human toxocariosis. Mature *Toxocara* worms live in the intestine of definitive host and by disposing of the eggs, contaminate the environment (2). In the life cycle of *Toxocara*, humans, birds and rodents are paratenic hosts. Infection in human occurs by ingestion of eggs; the main routes of infection are via contaminated hands by direct contact with puppies of dog or cat, contaminated soil, water and vegetables and ingestion of raw animal tissues infected with parasite larvae (3).

The risk of infection is higher in the first decade of life, due to more geophagic behavior and use of public parks by children. Most of the infected humans are asymptomatic and probably morbidity of the infection depends on parasite burden and immune response of the host (3, 4). Although human infections with *Toxocara* spp. are typically asymptomatic, larval migration into the internal organs via the blood can cause various clinical syndromes including visceral larva migrans (VLM), ocular larva migrans (OLM), covert toxocariosis (CT) and neurological toxocariosis (NT). The manifestation of symptoms in human toxocariosis depends on multiple factors, including the affected organs and the magnitude of the infection (4, 5). The common clinical manifestations of toxocariosis may include dyspnea, cough, chest discomfort, asthma, skin itching, and gastrointestinal disorders (3, 6, 7).

In humans, parasites cannot mature to the adult stage; so, examination of stool for parasite and eggs is not useful for diagnosis of infection. Biopsy is the gold standard for diagnosis of human toxocariosis, but it is a difficult method; thus, serological methods are the diagnostic mainstay. The enzyme-linked immunosorbent assay (ELISA) and indirect fluorescent antibody test (IFAT) are among the most common serological tests for diagnosis of toxocariosis (8, 9).

Iran is an endemic area for *Toxocara* species. Many studies in different parts of the country indicated the infectivity of dogs and cats with *T. canis* (10, 11) and *T. cati* (12, 13); as well as soil contamination of public places with parasites eggs (14, 15). In addition, several serological studies, carried out in different regions of Iran for evaluation of *Toxocara* infection in at-risk populations, emphasize the importance of this infection, especially in children (16–18). Nevertheless, in Aras Free Zone (Jolfa City), Northwest Iran, despite presence of roaming dogs and cats, information is lacking about this infection.

Therefore, this study aimed to determine the sero-prevalence of *Toxocara* spp. infection, associated with some demographic criteria and potential risk factors, among children 4–12 yr old in this area.

## Methods

### Study area

**Fig. 1:**
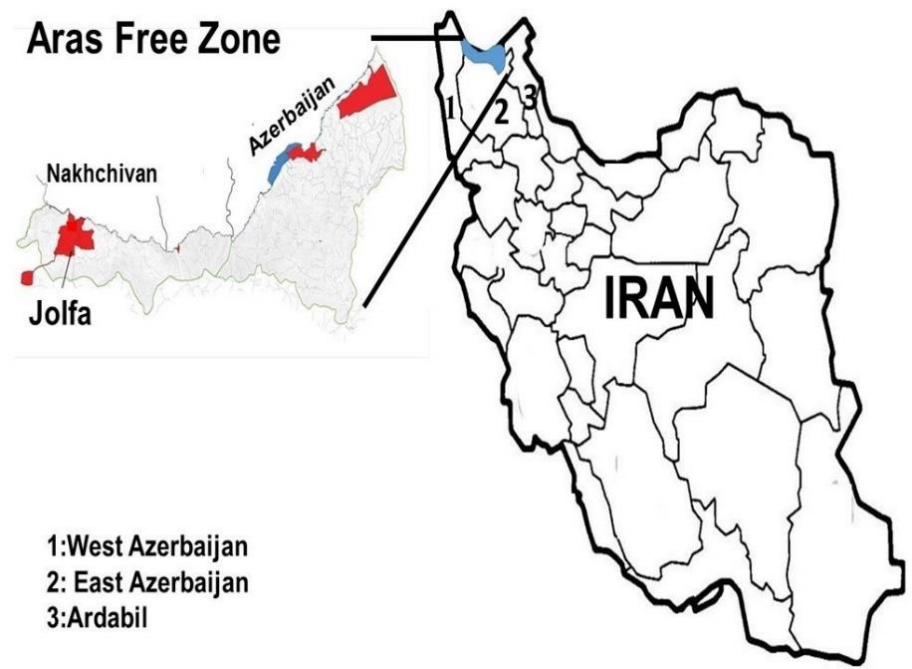
Location of Jolfa City in Aras Free Zone, Northwest Iran

This descriptive cross-sectional study was carried out in Jolfa City, as the center of Aras Free Zone, Northwest of East Azerbaijan Province. Jolfa City is located at the height of 710 meters above the sea level and at the North of the Kiamki Dagh Mountain and the South region of Aras River. This county is limited to Marand and Zaraghan from the South, the Kaleybar County from the East, the West Azerbaijan Province from the West and the Republic of Azrbaijan and Armenia from the North (Fig. 1). Jolfa City has semi-dry and semi-cold climate. Its annual rainfall is about 225 to 400 ml per year and average temperature is about 15 °C.

### Sampling

Sampling was carried out on children aged 4–12 yr old in urban and rural areas of Jolfa City. Overall, 514 blood samples were collected from children referring to two hospitals and four health centers and laboratories of this city, from May 2018 to Feb 2019.

To prepare the samples, first written permission was obtained from the parents of every participant. Then a questionnaire was completed intending the information about the demographic characteristics of child sex, age, mother educational level and also potential risk factors of toxocariosis, including history of close contact with dogs or cats and with soil, history of asthma or consistent fever, and place of the residency (rural or urban). Then, 2 cc of venous blood was taken from each child.

### Laboratory tests

The blood samples were transferred to the laboratory and after centrifugation at 5000 rpm for 5 min, the sera were removed and stored at −20 °C until further use for serological test. Detection of anti-*Toxocara* antibody in every serum was performed using an enzyme-linked immunosorbent assay (ELISA) kit (Nova Tec, GMBH, Germany) according to its instruction. An absorbance reading greater than 0.49 optical density (OD) units was considered as a cut off for seropositivity. Positive and negative controls were also used in each run. The kit sensitivity and specificity were stated as >95 percentages. The reading was acquired using a microplate reader (BioTek, United States), set at an absorbance level of 450 nm.

To avoid false-positive results and probable cross-reactivity with human ascarid nematode (*Ascaris lumbricoides*), formalin-ether concentration technique was performed on stool samples of those seropositive children that contributed during following up (eight individuals). Among them, for four children triple sampling was provided and for four other one's single samples.

### Ethical approval

This study was approved by the Ethics Committee of Tehran University of Medical Sciences, Tehran, Iran (IR.TUMS.SPH.REC.1397.097). A written informed consent was obtained from parents of the children before blood sampling.

### Statistical analysis

The SPSS statistical software, ver. 16 (IBM, NY, USA), was used for analysis of the results. Prevalence was described as relative frequency with 95% confidence interval (95% CI). Differences between categorical variables were analyzed by χ ^2^ test. The risk factors were tested univariate by logistic regression with adjustment for confounders.

## Results

Out of 514 participants (children 4–12 yr old) enrolled in this study, with a mean age of 8.8 yr old (standard deviation: SD ± 2.45); the overall prevalence of anti-*Toxocara* IgG antibodies was 2.3% (12/514). Seropositivity rates concerning different criteria are presented in Table 1. To compare the current results with similar results from other parts of Iran, the previous reports have been summarized in Table 2.

**Table 1:** Seroprevalence rates of *Toxocara* infection among children (4–12 yr old age) referred to the medical centers of Aras Free Zone (Jolfa City), Northwest Iran, according to socio-demographic characteristics (n=514)

*** Characteristic ***	*** Number examined ***	*** Seropositive number (%) ***	*** P-value χ ^ 2 ^ test ***	*** P-value in logistic regression ***
Sex				
Male	268	5 (1.9)	0.56	0.1
Female	246	7 (2.9)		
Age(yr)				
<7	105	6 (5.7)	0.02	0.03
≥7	409	6 (1.5)		
Mother education level	377	10 (2.7)	0.6	0.8
Illiterate & primary school	42	1 (2.4)		
High school	95	1 (1.1)		
College and higher Pet (dog and cat) contact				
Yes	51	3 (5.9)	0.1	0.19
No	463	9 (1.9)		
Soil contact				
Yes	116	5 (4.3)	0.15	0.21
No	398	7 (1.8)		
Pet or soil contact				
Yes	139	8 (5.8)	0.004	0.005
No	375	4 (1.1)		
Asthma				
Yes	10	0	0.78	0.98
No	504	12 (2.4)		
Persistent fever				
Yes	19	1 (5.3)	0.36	0.38
No	495	11 (2.2)		
Place of residency				
Urban	389	8 (2.1)	0.48	0.49
Rural	125	4 (3.2)		

**Table 2:** Seroprevalence of human *Toxocara* infection reported by previous studies in different parts of Iran

*** First author/Ref ***	*** Publication year ***	*** Province of study ***	*** Study population ***	*** Sample size ***	*** Seropositivity (%) ***	*** Technique ***
Sadjjadi/ (18)	2000	Fars/Shiraz	School-children	519	133 (25.6)	ELISA
Rokni/(43)	2000	Several pars of Iran	VLM cases	10	10 (100)	IFA
Fallah/ (16)	2003	Hamadan	School-children	544	48 (5.3)	ELISA
Akhlaghi/ (17)	2006	Kermanshah	Children	260	22 (8.6)	ELISA
Nourian/(42)	2009	Zanjan	School-children	810	22 (2.7)	ELISA
Sharif/ (29)	2010	Mazandaran/Sari	School-children	1210	297 (25)	ELISA
Alavi/ (30)	2011	Khuzestan/Ahwaz	Children 6–15 yr	203	4 (2)	ELISA
Garedaghi/(28)	2013	East-Azerbaijan	Children 0–15 yr	336	99 (29.5)	ELISA
Hosseini-Safa/ (27)	2015	Isfahan	Children 5–15 yr	427	6 (1.39)	ELISA
Momeni/(40)	2016	Urmia	Children, young 2–20 yr	397	12 (3)	ELISA

Population sample of current study comprised 268 males and 246 females. According to Table 1, 1.9% of males (5/268) and 2.9% of females (7/246) were infected with toxocariosis. However, there was no significant relationship between gender and *Toxocara* infection.

Results by chi-square test showed that anti-*Toxocara* seropositivity was associated with the age of children (*P=*0.02). In addition, in logistic regression analysis for different criteria, age showed significant difference (*P*=0.03) with the seropositivity. Therefore, children under 7 yr old were statistically more infected (5.7%) than those of ≥ 7 yr old (1.5%).

The rates of seropositivity in the children whose mothers had education levels of illiterate or elementary, high school, and college or higher levels were 2.7%, 2.4% and 1.1%, respectively. However, these differences were not statistically significant. Respect to exposure to potential contamination sources, although for both criteria of close contact with domestic animals (dog or cat) and contact with soil, being in touch was associated with higher rates of infection, the differences were not statistically significant (Table 1).

However, the rate of seropositivity in children who had contact with either pet animals and/or soil was 5.8%; and for the children who did not have such exposure was 1.1%.; this difference was significant with both Chi-square test (*P*=0.004) and logistic regression analysis (*P*=0.005).

There was no statistical difference between the presence of symptoms of either asthma or persistent fever and *Toxocara* seropositivity. Additionally, no association was found between residency in rural or urban areas and presence of anti-*Toxocara* antibodies.

To ensure about avoidance of probable false-positive results and cross-reactivity with other ascarids, stool examination were carried out for eight seropositive children whose samples collection could be possible during the following up. All these samples were negative for *A. lumbricoides* eggs and eggs/larvae of any other intestinal helminthes detectable by formalin-ether concentration technique.

## Discussion

Since treatment of toxocariosis is often ineffective, and no safe and effective vaccine is yet available, it is critical to make efforts to decrease the transmission of infection to reduce the serious manifestations of the disease (19). Finding the associated risk factors in every population of endemic areas is helpful in the management of preventive strategies. This effort requires speculation on the distribution of infection in the target area and its association with different potential socio-demographic criteria. Diagnosis of human toxocariosis is commonly based on serological tests such as ELISA to detect anti-*Toxocara* antibodies (20, 21). In this study, using ELISA test the sero-prevalence of *Toxocara* infection and associated risk factors were evaluated in children of Aras Free Zone in Northwest of Iran which is an area with lack of any previous information on toxocariosis.

Sera, collected from 514 children aged 4–12 yr old attending to some medical centers in the study area, were tested and the seropositivity was determined. Overall, 12 cases (2.3%) were found seropositive for anti-*Toxocara* IgG antibody. Positive serological tests of *Toxocara* are particularly common in developing countries. Extent of seropositivity of antibodies against *Toxocara* (based on ELISA test) are variable in different studies in the world. There are reports of 2.4% in Denmark (22), 10.9% among children of Jordon (23), 12.9% in Turkey (24), 23.5% in Serbia (25), and 92.8% in La Reunion (Indian Ocean) (26). Previous serological studies in Iran indicated different seroprevalence rates from 1.39% in Isfahan (27) up to 29.5% in Tabriz (28). Other similar studies in Iran have reported seroprevalence rates of 5.3% in Hamadan (Northwest of Iran) (16), 8.6% in Kermanshah Province (West of Iran) (17), 25.6% in Fars Province (South of Iran) (18), and 25% in Sari City (North of Iran) (29). These variations among children in various parts of Iran and other areas of the world might be related to sample size, transmission routes, climatic conditions of each study area and socio-cultural factors.

Large-scale studies are indicating no association between gender and toxocariosis (30, 31). In our study, although the infection rate was higher in girls than boys were, there was no significant correlation between gender and seropositivity. In accordance with these results, in Hamadan, western Iran, on children of 1 to 9 yr old, no relevance was found between age, sex, and place of residency and anti-*Toxocara* seropositivity (16). However, in some studies, the associations between gender and infection rates have been reported to be significant. For example, in a study performed in Trinidad, infection with this helminthic parasite had sex tendency with higher rate in boys. Seropositivity, in that study, in boys and girls were recorded as 58% and 41%, respectively (32). Differences in seropositivity between the two sexes may be due to the differences in gender-specific behavior and type of games in girls and boys.

Statistical analysis of present results revealed significant association between age of the children and seropositivity. Children under 7 yr old were significantly more infected than those of older children (*P*=0.02). Therefore, young age (<7 yr old) was considered to be the prominent risk factor of infection. These results are in agreement with those reported in Brazil who found that children of 5–8 yr old were more likely to be positive for *T. canis* VLM (33). Another study in India showed a higher prevalence in groups 1 to 10 yr of age, reported to be due to pica and contact with puppies (34). In West Indies, the prevalence rates of infection were related to age, and the age group of less than 5 yr old was more at the risk of infection acquisition (35). In contrast, in the north of the Mexican Valley, the older group (12–16 yr old) had higher rate of seropositivity (36). In general, in the world toxocariosis is mostly considered as a common parasitic infection among children which may be due to childhood activities and higher contact of this group with the resources of contamination (37, 38), as well as their lower immunity against the infection.

Based on the results of this study, although with increasing the level of education of children’s mothers the rate of anti-*Toxocara* seropositivity decreased, however, the differences among the different groups were not significant. That might be due to the sample size effect. However, in the USA (39), Northwest of Iran (40) and Southeast Brazil (41) reported significant correlation between educational level of parents and rate of seropositivity. Increase of knowledge has positive impacts to decrease the risk of the infection. Lower education levels of parents are often associated with lower socioeconomic status that reinforces the risk of *Toxocara* infection in their children.

In the present study, due to the sample size effect, no association was found between *Toxocara* seropositivity and pet animal contact or soil contact once each of these risk factors analyzed solely. In Kermanshah (17) and Shiraz (18) also no significant correlation was reported between contact with soil and seropositivity. In contrast, in Serbia (25) significant correlation was reported between contact with soil and seropositivity. In our study, when the two risk factors of contact with pet animal and/or soil were considered together, and the pooled data were analyzed, the results demonstrated the importance of these resources as potential risk factors for the acquisition of infection.

Based on the current results, there was no statistically significant correlation between both factors of history of asthma or persistent fever, and the seropositivity. Additionally, the rate of seropositivity in children of urban and rural areas were 2.1% and 3.2%, respectively, without significant difference. Lack of association between place of residency and *Toxocara* seropositivity have also been found in the studies in Ahvaz, Khouzestan Province, south-west Iran, (30), and Isfahan Province, south Iran among school students (27). While, on the contrary, in the study of Zanjan Province higher infection rates was reported in children of rural areas (42). In the present study, contact with source of contamination i.e. pet animals and/or soil, was relevant to the infection rate rather than residency in urban or rural areas. Therefore, it seems differences on the effect of place of residency in various studies is more probably due to the differences in the lifestyle, contamination of the environment with parasites eggs, and extend of residents exposure with contaminated resources, especially children. In our study, the main limitations were lack of checking for clinical signs of the participants, and not being able to conduct a following up effort on the infected children. The strength of the study is analysis of *Toxocara* infection in children and its association with important risk factors in a city without previous information.

## Conclusion

Both girls and boys are at risk of *Toxocara* infection in the study area. Younger age of childhood and contact with sources of infection were important associated factors. More probably, in addition to the effect of children age and their contact with pet animals and/or soil, additional criteria are involved in the initiation of infection. Further assessments of toxocariosis in relation with involvement of patients with hyper-eosinophilia, epilepsy, and schizophrenia is recommended. The findings of the study may be a warning for attention of health authorities for screening the infections among children and other population at risk and designing of programs for the prevention of infection.

## Ethical considerations

Ethical issues (Including plagiarism, informed consent, misconduct, data fabrication and/or falsification, double publication and/or submission, redundancy, etc.) have been completely observed by the authors.
